# An Experimental Evidence on Public Acceptance of Genetically Modified Food through Advertisement Framing on Health and Environmental Benefits, Objective Knowledge, and Risk Reduction

**DOI:** 10.3390/ijerph18105264

**Published:** 2021-05-15

**Authors:** Syed Hassan Raza, Umer Zaman, Paulo Ferreira, Pablo Farías

**Affiliations:** 1 Department of Communication Studies, Bahauddin Zakariya University, Multan 66000, Pakistan; hassansherazi@bzu.edu.pk; 2 Endicott College of International Studies, Woosong University, Daejeon 34606, Korea; 3 VALORIZA—Research Center for Endogenous Resource Valorization, 7300-555 Portalegre, Portugal; pferreira@ipportalegre.pt; 4 Department of Economic Sciences and Organizations, Polytechnic Institute of Portalegre, 7300-555 Portalegre, Portugal; 5 CEFAGE-UE, IIFA, University of Évora, 7000-809 Évora, Portugal; 6 Departamento de Administración, Facultad de Economía y Negocios, Universidad de Chile, Santiago 8330015, Chile; pfarias@fen.uchile.cl

**Keywords:** genetically modified food, food security, food innovation, advertisement, attitude, acceptance, knowledge, message framing, perceived risk, science literacy model, cognitive miser theory

## Abstract

Owing to the emerging challenges on global food security and the decade of controversies over genetically modified food (hereafter GMF), the present study aims to explore the effects of advertisement framing on health and environmental benefits, sources of perceived risk reduction, and domain-specific knowledge on the acceptance of GMF. The study conducted a quasi-experimental factorial 2 (advertisement message framing: health vs. environmental benefits) × 2 (expert endorsement: present vs. absent) between-subject design involving 300 adult participants from Pakistan. Using a multi-group structural equation model, the four conditions were assigned to each participant group (*n* = 75) to test the hypothesized relationships. The quasi-experiment results suggested that the advertisement messages (ad-framed) incorporated with the health and environmental benefits, as delineated by experts, can be a viable communication strategy in developing effortless cognitive cues towards GMF acceptance. The pioneer findings validate the significant efficacy of advertisement messages (ad-framed with expert opinions) in reducing perceived risk through augmented objective knowledge that activates the mechanism of favorable development of attitude and acceptance of GMF. The study findings offer strategic directions to policymakers, marketers, and food technologists in raising greater awareness and acceptance towards GMF products.

## 1. Introduction

Ending food insecurity and malnutrition through eco-friendly means of agriculture production is a persistent worldwide priority [1,2]. The present worldwide strategy for prevailing food, nutrition, and eco-friendly production has not been able to sufficiently address the global challenges to ensure development toward food and environmental security [3,4]. Innovative high-impact food technologies, such as genetically modified food (hereafter GMF) and biotechnology, now provide prospects for enhancing food production and increasing food quality and nutritional value [5]. For instance, food staple (e.g., rice, wheat, etc.) varieties are essential, which are capable of persisting and even flourishing in a variety of hastily varying and risky ecological circumstances [6]. Hence, these innovative food technologies, such as GMF, enable the public to eat foods with rich nutritional values [7]. At the same time, this is a substantial task, resting on the public acceptance of these environmentally safer and nutrient-rich GMF food innovations, generally produced through genetic manipulations of novel varieties of staple crops (e.g., rice, wheat, etc.) [8].

Addressing the growing concern for global food security and the challenge to feed nine billion inhabitants of this planet by 2050, GMF has been a recent advancement of food-based technologies [9,10]. Besides, more than two billion people globally are combating malnutrition due to vitamin and mineral deficiencies [11]. The situation of malnutrition among the people living in developing nations is a more severe problem. Therefore, the usage of GMF can provide healthier solutions that can reduce food insecurity and malnutrition considerably [12]. Despite the efforts of advocacy groups for GMF, its public image and acceptance have remained a critical concern. Hence, marketing-driven promotional campaigns can be useful to educate and reduce the public’s distrust towards GMF products [1].

Regardless of GMFs’ nutritional values and environmental benefits, the literature affirms the large controversies attached to GMF, based on perceived risks and limited public knowledge [1,3]. With the advent of scientific innovations, the global food industry has adopted the newly innovated food generics to address issues of food sovereignty and security [1,2]. GMF products have saturated the worldwide market and continued to rapidly expand as an industry owing to the technically demonstrated innovations in GMF [13]. Given that farmers are increasing their crop production and reducing their fertilizer usage, the public can get cheaper nutrient-rich food. In a similar vein, using such food technologies (e.g., GMF), manufacturers can decrease their production costs and provide eco-friendly GMF. Until now, the public can be considered as the direct beneficiaries of GMF technology [14]. In this way, the scientifically demonstrated inventions in GM technology can unswervingly provide an advantage to the public as well as society [15]. For instance, GMF can provide healthier nutritious values to consumers, diminish food security threats, and increase environmental benefits to society [16]. Owing to GMF’s role in tackling food security issues, health, and environmental benefits, GMF producers, as well as many nations, have prioritized promoting GMF consumption [16].

Even though some governments have prioritized GMF adoption, there have been many instances wherein consumers have raised social and health concerns about GMF usage. Thus far, opposition in many nations, e.g., European nations, has raised concerns related to the susceptibility of GMF food usage and imports [17]. On the other hand, some GMF consumers use GMF without knowing it, based on assumptions that GMF has never harmed anyone. Therefore, it is quite evident that public perceptions about GMF can influence the degree of its acceptance [18,19,20]. The apparent elucidation of this phenomenon is that most consumers rely on expert opinions, media reports, and advertisements to acquire knowledge about GMF products’ benefits, as well as risks [21]. Hence, consumers tend to seek objective knowledge about innovative products’ usage, such as GMF, which leads them to information dependency [16,22,23]. Generally, experts’ opinions that are accessible to consumers are based on scientific product benefits and sometimes include risk factors as well. Another accessible source to develop perception is advertisements of products; however, sometimes, consumers remain susceptible of advertisements as paid content [24]. Therefore, media reports are a substantial source of information when consumers select their behavior.

Hence, consumers require safety-related objectives and pertinent knowledge about scientifically innovated technology, such as GMF, to diminish their risk perceptions [25,26]. This is consistent with science literacy theory [27], in that a simple description of scientific technology implications is essential for individuals to acquire such innovations [28]. Therefore, objective knowledge is vital in shaping consumers’ acceptance of GMF. In the context of Pakistan, which is characterized by a comparatively high-uncertainty culture and is inclined to be more risk averse, GMF products are quite new and many manufacturers have recently started moving towards GMF production [14]. Thereby, perceptions about innovative products may induce more risk factors, such as psychological, physical, and performance risks. Due to potential concerns of Pakistani consumers’ about GMF, including (1) the health safety of GMF usage, (2) the potential threats of GMF usage to the environment, and (3) the cultural preference for natural food, policymakers and the GMF industry may face challenges in promoting GMF in Pakistan. The current experimental research seeks to examine possible communication strategies to overcome these challenges. On the other hand, usage of GMF can address the food security issues emerging in Pakistan due to productivity issues owing to climate change and minimal usage of advanced agricultural or food technologies. Therefore, an understanding of how acceptance of GMF usage can be enhanced through effective communication strategies is required [19].

In order to increase acceptance of innovative products, such as BFF, researchers have identified a variety of execution strategies utilized in advertising that vary from the use of information appeals, emotional appeals, and neural process evoking [29,30]. The studies were intended to investigate assorted feelings, such as rationale appeals [31], emotional appeals [32], and informational [22] as the most common appeals. Hence, the literature has found that the use of these adverting appeals is beneficial in influencing an individual’s attitudes towards innovative products. The theoretical perspective of the science literacy model (hereafter SLM) also supports the role of informational content in altering consumer behavior through improved knowledge and a reduction of perceived risk [33]. However, in circumstances in which human and environmental wellbeing may be at risk, individuals always require trustworthy information that can assist them in evaluating the inevitability, intensity, and imminence of the hazard [34].

Ergo, past theories, for instance, the heuristic-systematic information processing model (hereafter HSM), also explained that this mechanism does not only depend on the content of the ad message; it also relies on the quality of the message source [35]. To exemplify this, individuals’ processing of the acquired information through an advertisement involves risk assessment based on the credibility of the sender and presentation cues (e.g., ad-frame). In this way, individuals balance apparent threats and benefits to decide whether to use the product in question. On the other hand, past research also recognized that psychological, physical, and performance-related risk perceptions are paramount in determining consumer behavior [36,37]. In the case of GMF, recent literature also noted that social elements, such as individuals’ preferences for natural food, can influence the acceptance of GMF products [31]. However, minimal effort has been expended to ascertain how expert viewpoints incorporated into ad messages impact one’s response to GMFs. Research investigating the efficiency of expert incorporated messages about the scientific agreement has emphasized issues, such as the environment [38]. The findings of these studies suggest that underlining experts’ opinions in messaging on a specific issue can reduce the uncertainties about that issue by providing action cues and cognitive shortcuts [39]. There is a plethora of literature supporting that the public trusts scientific experts to acquire precise and trustworthy knowledge on particular issues of a scientific nature [40]. Similarly, it is evident that domain-specific knowledge when provided by domain-specific experts is regarded by the public. However, there is a knowledge gap, as past studies have not tested these conceptual linkages in an integrated model yet. The current study is designed to address the question of whether the combination of expert opinions and issues affect the mechanism involved in the acceptance of GMF compared to informational content alone. Regarding the development of a promotion campaign of GMF in Pakistan, this would certainly address the following strategic question: What would be an effective way to persuade the public? The findings of this study shed light on these pertinent issues and provide future directions to design an effective promotional campaign. Details of this phenomenon and conceptual linkages are discussed in the next sections.

## 2. Literature Review and Theoretical Underpinning

### 2.1. Integrating Science Literacy Model (SLM) and Cognitive Miser Theory (CMT)

Prior literature on science communication advocates that in case of promoting debatable and innovative technologies, such as GM food, providing scientific literacy is an effective communication strategy [36]. This will enhance the acceptance of such technologies among the public by decreasing their risk perceptions. However, a plethora of literature has affirmed that people rely on cognitive shortcuts, which serve as perceptual filters, such as value predispositions and heuristic cues [41,42]. The findings of previous studies also indicated that cognitive shortcuts forecast an increase in positive attitudes towards controversial or debatable technologies. In this regard, the literature has identified several value predispositions, such as message credibility/endorsement and heuristic cues, for example, perceived value, cost, and perceived risk [43].

**Figure 1:**
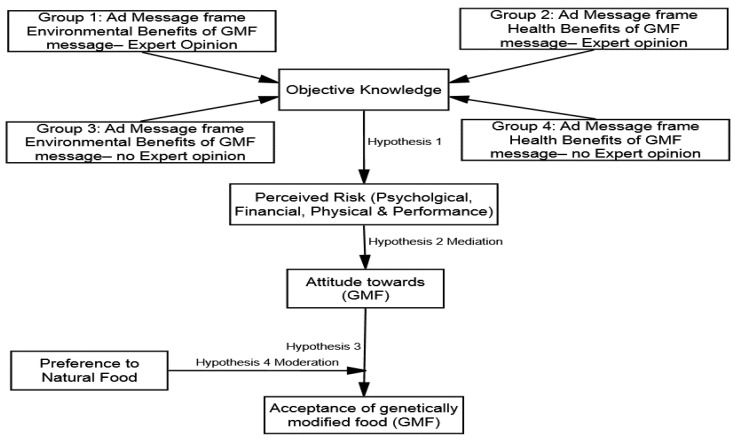
Conceptual model of the study.

These cognitive shortcuts along with knowledge can offer a perceptual filter through which individuals formulate attitudes towards GMF [44]. This is in line with the cognitive miser theory (hereafter CMT), which states that individuals usually depend on cognitive shortcuts to formulate attitudes about debatable technologies [45]. Once individuals have a positive attitude formulated toward the usage of the GMF as a result of perceived risk reduction, acceptance of GMF is also increased. However, the literature has also identified certain social factors, such as a preference for natural food, that may also determine the acceptance level among individuals [46]. Drawing an analogy of the past theories of SLM and CMT, a conceptual model (see Figure 1) is proposed, illustrating that advertising message frames/appeals would help to increase one’s objective knowledge. Consequently, improved objective knowledge and advertisement messages would provide cognitive shortcuts to individuals. These cognitive shortcuts would reduce the perceived risks (e.g., psychological, physical, and performance) and improve the perceived value of GMF, which would mediate the effects of different types of objective knowledge on consumers’ attitudes towards GMF. Therefore, this study identifies four main factors to explain the acceptance of GMF: (1) objective knowledge, (2) perceived risk reduction based on cognitive shortcuts developed as a result of improved objective knowledge, (3) attitude towards GMF, and (4) the social factor of a preference for natural food.

### 2.2. Objective Knowledge

Two approaches prevail in the literature regarding examining the influence of scientific knowledge on public acceptance of scientific and technologically advanced products [47,48]. One approach looks at the association of objective knowledge (i.e., domain-specific or subject knowledge) with public acceptance and behaviors towards science and technology [49]. In contrast, the second approach highlights the association of general science knowledge with public acceptance and behaviors towards science and technology [40]. Objective knowledge can be described as the individual’s actual knowledge about the product in question, while subjective knowledge refers to the perception of an individual of how much he/she knows about a product [50]. However, some of the literature suggests that objective knowledge influences consumers’ decisions about technology. For example, the literature has identified that public domain-specific (objective) knowledge positively influences the acceptance of genetically modified rice affected by consumers’ objective knowledge. These findings imply that the public relies upon domain-specific knowledge to make decisions concerning technology-based products [51]. Hence, objective knowledge can reduce the risk perception about GMF. Meanwhile, studies suggest that mass media content, such as advertising, containing domain-specific knowledge could be the primary source of prompting GMF for marketers [52]. Therefore, this research aims to understand the role of objective knowledge development through advertisement message framing.

### 2.3. Advertisement Message Framing

Advertisement message framing refers to ad messages that are designed to attain particular meanings through the presentation of facts in messages [53]. Advertisement framing has been widely used by advertisers to present an ad-message in a way that impacts how receivers decode such a message. Such an advertisement message frames the facets of the issue in question to regulate its essence by using strategic verbal and non-verbal advertising tools, such as information and the source of information [54]. Rational advertising framing stems from conventional information processing theories, which posit that an individual makes a rational and cogent choice about the product, mainly by viewing its benefits, such as quality, worth, or presentation [29,30]. Accordingly, rational advertising appeals are described as the extent to which an advertisement concentrates on rational reason to motivate the person [55]. Rational appeals include exhaustive information or convincing and logical arguments. The consumer values advertisements that have more persuasive content, which leads to the consumer making a favorable assessment [56]. These aspects of the advertisements (ad appeals) prime the development of a positive attitude and improve the advertising value. Research opined that rational appeals are based on logic to highlight the characteristics, eminence, problem-solving capability, and performance of the product [57].

Hence, an advertising message framed about a product/service benefit, such as attributes or utility, can be effective [58]. In this regard, the benefits (i.e., health and environmental) incorporated in advertising appeals can communicate objective knowledge to the public and strive to make the public believe that the GMF product is a healthier choice. However, the celebrity endorsement technique is also widely used in advertising to improve source credibility [59]. Consumers value the advertisement message if it not only gives accurate facts about the products but also comes from a credible source [60]. The literature indicates other critical factors, such as involvement and concerns, in determining consumers’ motivation to act. For example, someone who is conscious about the environment may show more involvement in advertisement messages framed to provide environmental benefits of products.

### 2.4. Perceived Risk Reduction

Scholars [44] have described perceived risk as a two-facet variable, such as uncertainty and adverse outcomes, which makes it greatly pertinent to investigation concerning innovative goods, for example, GMF. In a similar vein, some studies [61] have indicated two formative dimensions of perceived risk, including (1) dread and (2) unknown. The dread risk is devised from a possibly risky activity, for instance, when the use of new technologies is considered a hazard to life, or possibly unsafe for the public. Conversely, unknown risk originates when individuals are not aware of or unsure about the use of potential outcomes of an activity, such as the use of new technology. There is abundant literature that supports that the use of food produced as a result of modern technologies, such as GMF and biotechnology, is viewed as an unknown risk [40]. Similarly, owing to the potential harm to human and environmental health, use of these technologies has been conceived as dread risks [62].

The notion of “perceived risk” in such circumstances is associated with limited awareness or knowledge, as the outcomes of new or controversial product usage and the likelihood of those aftermaths in reality happening are greatly unknown [61,62]. Numerous distinctive kinds of risk perception related to the acquisition and new or controversial product usage have been recognized, comprising (1) cost or economic, (2) performance, (3) physical, and (4) psychological (e.g., Jacoby and Kaplan, 1972). Past studies have shown that usually consumer appraisals of risks as well as benefits function as an essential element that regulates the consumer’s attitude towards GMF [47].

The current research includes four facets of risk reduction perception: financial, psychological, performance, and physical. These four perceived risks are crucial for deliberations about GMF, especially in the context of Pakistan, because the extent of uncertainty towards the adoption of innovative products has been found to be higher among Pakistanis. Physical and performance risks deal with the functional aspects of GMF, psychological risks deal with the emotive aspects, and financial risks create an economic component of risk [61]. Owing to the limited acquisition and knowledge regarding genetic modification procedures adopted for food technology, these four risk factors are critical in determining attitudes towards GMF.

However, reducing the risk perceptions of people through improved knowledge may result in a positive attitude towards GMF usage. Ergo, the study conceptualized perceived risk (psychological, physical, etc.) as individuals’ perceived possibilities and severity of an impending risk based upon past literature’s descriptions of the risk perception as persons’ “subjective judgment about the likelihood of negative occurrences” [26]. This study investigates whether individuals’ perceived risk related to GMF would diverge when they are exposed to ad messages framed to delineate the benefits of GMF. This phenomenon is assumed based on SLT and CMT, in that when individuals are exposed to ad messages framed to support GMF food, individuals come to comprehend the benefits of this food technology. This study probes whether risk perceptions towards GMF are reduced after exposure to objective knowledge-based advertisement messages.

Based on previous literature, when information is offered with rationalized facts about new technology-driven products, viewers comprehend multifaceted technical procedures better [52]. In contrast, psychological theories (e.g., HSM) presume that when these factual details are presented with expert opinion, viewers may perceive the adoption of technology-driven products with less doubts and uncertainties [35]. Furthermore, dual information processing models imply that people are much more mindful about the usage of products with higher risks [63]. They appraise the given information more carefully and critically; they consider the message content, credibility of the source, and usefulness of the product. This theoretical explanation clarifies this phenomenon, in that information coming from a credible source is an influential feature of message framing that can help people to evaluate risk. The communication literature has also noted that if ad messages are integrated with experts’ (scientists) viewpoints advocating the health or environmental benefits of a novel product, an individual processes such messages more effectively [64]. Because they get cognitive cues and process such information more effectively, they reduce their risk perceptions towards novel products. Therefore, the study proposes that with improved objective knowledge, people have fewer uncertainties about the use of GMF; however, expert opinion plays an influential role in decreasing risk perception. Hence, we hypothesized that:

**Hypothesis** **1** **(H1).** *Knowledge will have a positive influence by lowering the risk perception of GMF for ad messages framed with experts—those with either health benefits or environmental benefits—compared to those without experts.*

### 2.5. Attitudes towards GMF Usage

Attitudes can be determined by the aggregate of the individual’s beliefs that they uphold towards displaying a particular behavior, subjective to the appraisals of the upheld beliefs [65]. At large, those actions or behaviors that are supposed to result in an advantageous consequence have favorable attitudes connected with them [66]. In contrast, actions or behaviors that are supposed to result in undesirable consequences have unfavorable attitudes connected with them. In the instance of GMF product usage, the consequences connected with consuming them are generally vague among common people as both harmful and expedient consequences can be found in national and international media about innovative products [67]. In general, a common person can have more uncertainties about the adoption of such innovative products based on the uncertain attitudes formed because of unknown or dreaded outcomes related to health or environmental concerns [68]. However, SLM argues that by providing objective knowledge or educating the common person through informed and relevant persons, such as experts, people can become aware of accurate and reliable information [40]. Subsequently, this would lessen their perceived risks associated with the use of a particular product, such as GMF, and result in improved attitudes. To this end, communication messages through a known source can function as a platform to provide objective knowledge to people regarding GMF. However, certain beliefs (perceptions) are the underlying mechanism in developing a positive attitude towards GMF usage because improved knowledge can reduce risk perceptions and consequently result in a positive attitude. Therefore, we propose that perceived risks mediate the effects of knowledge about GM on attitudes and hypothesize that:

**Hypothesis** **2** **(H2).** *Perceived risk reduction will mediate the relationship between objective knowledge about GMF and attitudes towards GMF.*

### 2.6. Acceptance of GMF Usage

Scholars [47,69] have underlined several aspects of acceptance of controversial technologies or products, such as GMF, and identified three aspects of acceptance: (1) socio-political, (2) community, and (3) market. The literature shows that researchers have identified suitable and rationalized antecedents of technology acceptance during their research [44]. For instance, the public, generally at the socio-political level, accepts technology easily as it commonly does not deliberately harm the public, such as health or environmental hazards. Conversely, when acceptance is demanded at a personal level, they start identifying the complications involved in accepting technology, such as GMF, and this phenomenon is known as community acceptance [70]. However, at the market level when an individual is asked to adopt or consume a technology-based food product, it must be evaluated through his/her own situational and perpetual factors. In this standard, adoption encompasses one’s intentions towards a particular technology [71]. From the perspective of technology acceptance, such as GMF, research has affirmed that discrepancies are expected between these three levels of acceptance, and one’s acceptance in terms of adoption would depend on attitudes, which are guided by risk evaluation of the usage [72]. Drawing on past literature, research has shown that the aspect of discrepancies for personal (health concerns) and general (environmental concerns) acceptance would also be crucial in the case of GMF usage. Therefore, individuals would be concerned and desire greater risk reduction to form a favorable attitude that would guide their acceptance of GMF usage.

In this way, attitude plays an imperative role in the acceptance of technology, such as GMF, and attitudes towards GMF usage are likely to be influenced by risk perceptions of GMF [47]. Generally, people have uncertainties regarding the usage of GMF, as past research provides evidence regarding whether information can reduce perceived risks and promote the benefits related to GMF through improved objective knowledge [52,73]. However, dual information processing approaches have not been validated, for example, knowing whether people’s attitudes towards GMF varied after exposure to the different techniques of ad message framing or not. Most research in the past has focused on the benefits of GMF only, for example, GMF can increase the yield and nutritional content [74]. This research addresses the minimally researched aspect of the acceptance phenomenon regarding how greater attitude change based on risk reduction can improve the acceptance of the GMF.

Our assumption is based on the theoretical notion that individuals’ accessible knowledge and inevitability about a threat regulates how they will respond [40]. For example, the rational choice theory posits that individuals assess the likelihood of consequences after they analyze the latent benefits of the phenomenon in question [75]. In this standard, individuals evaluate risks and make predispositions (i.e., their attitude towards GMF) to make decisions (i.e., acceptance of GMF) predominantly ascribed on the provided subject-domain knowledge. Most of the past research investigating the efficacy of the information provided by experts has argued that people usually trust expert opinions to yield precise and credible knowledge [34,76]. The trust determination theory also featured the value of scientific expert opinion in the development of predispositions [77]. As such, the information coming from scientific experts on a specific matter can impede individuals’ uncertainties and help people to adopt a particular behavior. Similarly, CMT theory also provides a reasonable understanding of how people depend on cognitive shortcuts [45]. Consequently, messaging containing an expert opinion can influence behavioral patterns as people perceive that experts are a reliable source of information in their relevant domain [78]. Therefore, it ought to follow that people’s acceptance of GMF usage will depend on advertisements comprising expert opinions compared to others; hence, we hypothesized that:

**Hypothesis** **3** **(H3).** *Attitudes towards GMF will positively influence acceptance of GMF usage but more favorably for (a) health-framed messages and (b) environmental-framed messages from experts compared to those without experts.*

### 2.7. Preference for Natural Food

The contemporary era has observed an intensification in public aspiration for naturalness, mainly in the case of food consumption [79]. Ergo, naturalness concerning food production without involving any technological alteration is gaining popularity among the public [80]. Similarly, some societies, such as Pakistan, have traditional preferences for natural foods (known as desi food in Pakistan). Such kinds of preferences for naturalness are characteristics of several societies, as they wish to avoid the consumption of products that are not naturally produced and manipulated by technology use. The literature indicates that some individuals pay attention to the naturalness of a product instead of the ingredients or production quality of the food itself [46]. Therefore, individuals with such an inclination for natural food were found to retain greater risk perception towards food produced through technologically sophisticated methods, such as genetically modified or nano-food, in comparison with others [79]. For this reason, such individuals perceive that the benefits of naturalness overtake the benefits available from GMF. Thereby, this research postulates that a preference for natural food is a potential moderating factor determining the strength of the acceptance of GMF usage and attitude linkage; hence, we hypothesize that:

**Hypothesis** **4** **(H4).** *A preference for natural food inversely moderates the relationship between attitudes towards GMF and acceptance of GMF.*

## 3. Materials and Methods

### 3.1. Design, Participants, and Procedure

This study employed a quasi-experimental factorial 2 (advertisement message framing: health vs. environmental benefits) × 2 (expert endorsement: present vs. absent) between-subject design to test the proposed hypothesis and research questions. Based on this design, four groups of participants were exposed to a separate manipulation: Group 1: Environmental benefits of GMF narrated by an expert; group 2: Health benefits of GMF narrated by an expert; group 3: Environmental benefits of GMF narrated without an expert; and group 4: Health benefits of GMF narrated without an expert. To perform the factorial designs, the literature has suggested a minimum (*n* = 30) for each group [81]. Overall, the current research involved four groups. Therefore, a sample (*n* = 300) was gathered from Pakistani nationals. In this standard, each group was composed of 75 participants who were exposed to the separate manipulations, which is consistent with the recommendations of past literature [81]. The sample was approached using the help of volunteers who approached them during their routines (shopping, offices, etc.) to ensure that participants remained natural. Before starting data collection, consent regarding participation was given to those who agreed to fill in the questionnaire after viewing the relevant manipulation. This is in line with the quasi-experiment design, which has no strict requirement of randomization, and instead ensures participants remain in natural settings.

### 3.2. Instrumentation

#### 3.2.1. Selection of Stimuli

To execute this study, 4 advertisement stimuli were designed: two print advertisement messages containing the environmental benefits of GMF, one narrated by an expert and one without the presence of an expert, and two print advertisement messages containing the health benefits of GMF, one narrated by an expert and one without the presence of an expert, targeted to improve the objective knowledge of the participants. The Urdu (national language) was used in the advertisement messages; an English version of the stimuli is shown in Appendix A. A content and face validation procedure were adopted based upon the validity ratings of the experts regarding the stimuli used for the main study. This procedure was used to attain translational and face validity [82]. The experts evaluated the content of all four advertisements, constructed definitions and items, and their rating was computed [83]. The study used a three-item scale to test the manipulation based on the ad-stimuli feature. The participants were requested to record their responses on a “semantic differential scale” after reading the statement that a “genetic modified food advertisement is: (1) 1 = extremely informative, 5 = not at all informative; (2) 1 = extremely reliable, 5 = not at all reliable; and (3) 1 = extremely persuasive, 5 = not at all persuasive. The result revealed a significant mean difference based on the post-hoc ANOVA *t*-test. Higher mean values were reported by participants in the group that were exposed to the expert opinions (Mean_G1_ = 3.98, SD = 0.84 Mean_G2_ = 3.79, SD = 0.67) compared to those who were exposed to advertisement messages with no expert opinions (Mean_G3_ = 2.61, SD = 0.35 and Mean_G4_ = 2.34, SD = 0.54). Hence, the results confirmed the manipulation of the stimuli (t = 8.14; *p* ≤ 0.00). Furthermore, Levene’s test of variance was carried out to validate the difference between subjects exposed to different types of advertisements, which also revealed significant differences (F (276) = 21.79, *p* ≤ 0.001).

#### 3.2.2. Risk Perception Reduction

The dimension of the reduction of the psychological risk was measured using two reversed items adopted from the literature [47]: (1) “GM food will lead to new human health and environmental problems” and (2) “The thought of purchasing GMF makes me feel psychologically uncomfortable”. The dimension of the physical risk reduction was measured using the two items: (1) “My chances of getting food allergies are great if I eat genetically modified (GM) foods” and (2) “There is a good possibility that my body will accumulate toxicity if I eat genetically modified (GM) foods”. These were averaged to create a composite index, with higher scores indicating higher risk perception (M = 4.45, SD = 1.15, Cronbach’s α = 0.92). Furthermore, the performance risk reduction was measured using two items: (1) “GM food does not taste as good as it should” and (c) “GM food ingredients in food will lead to better nutrition”. Moreover, the financial/cost-effective risk reduction was measured using the two items (one reversed): (1) “GM food is costly food” and (c) “GM food is financially viable to buy.” The participants were requested to give a response regarding the statements using a 5-point scale (1 = strongly agree, 5 = strongly disagree). The average of all the items was used as the participants’ overall perceived risk reduction, as extracted from the literature [47].

#### 3.2.3. Objective Knowledge

This study used the six-item GMF knowledge scale from the literature [9,30] (e.g., “When it comes to GM food, I don’t know a lot”), which were measured on a 5-point Likert-type scale (1 = strongly agree, 5 = strongly disagree).

#### 3.2.4. Attitude towards GMF

The attitude towards GMF was measured after exposure to the stimuli using a four-item “semantic differential scale” adopted from the literature [40] using the statement that “Genetically Modified Food is” followed by the response bipolar 5-point: 1 = extremely good, 5 = extremely bad); 1 = extremely superior, 5 = extremely inferior; 1 = extremely favorable, 5 = extremely unfavorable; and 1 = extremely appealing, 5 = extremely unappealing. The average score of the four items was used as the participants’ attitude towards GMF.

#### 3.2.5. Preference for Natural Products

A preference for natural products was measured by four items adopted from the literature [46]. The participants were requested to give a response about the following statements using a 5-point scale (1 = strongly agree, 5 = strongly disagree): (1) “I prefer to buy natural products”; (2) “To me, the naturalness of the food that I buy is an important quality”; (3) “I prefer to avoid food products with additives”; and (4) “I do not mind paying a premium for natural products”.

#### 3.2.6. Acceptance of GMF Usage

The acceptance of GMF usage was measured using participants’ responses on a 5-point scale (1= “strongly agree”, 5= “strongly disagree”) based on the behavioral associations related to the use of GMF. The following three items were adopted from prior literature [40]: (1) “I am willing to eat genetically modified food products”, (2) “I am willing to purchase genetically modified food products”, and (3) “I am willing to serve my family genetically modified food products”.

#### 3.2.7. Control Variables and Demographic

Gender, educational level, locality, and age group were collected and treated as the control variables in this study. The demographic distribution of the data is shown in Table 1. The demographic analysis reflects the overall Pakistani demographic categorization. For example, in terms of age, 50.7% of the population largely ties with Pakistan’s age-wise demographic attributes. However, the limitation of these demographic variables is related to the educational level. Approximately 70% of the populations are literate in Pakistan; however, the current sample represents 89%. This occurred due to the restriction of the experimental design and researchers’ demographic access because most of the data were collected in urban areas that comprised mostly literate people.

## 4. Results

### 4.1. Descriptive and Demographic Analysis

Demographic analysis was conducted before proceeding with the descriptive statistics on SPSS 24, including (1) normality checks, (2) outliers’ analysis, (3) variance inflation (VIF) test for multiclonality, and (4) bivariate correlation through Pearson’s test. After analyzing the normality visual inspections, a bivariate analysis was performed (see Table 2) separately for all four groups of data (*n* = 75). A significant relationship was found among all variables in all four conditions. The VIF value was found below 10, thus confirming that there is no multicollinearity involved.

### 4.2. Confirmatory Factor Analysis (CFA)

Afterward, using structural equation modeling (hereafter SEM), this study performed multiple confirmatory factor analyses (CFA) on AMOS. This was done to inspect the (1) convergent and divergent validity, (2) structural and measurement model fitness, and (3) inferential statistics. Firstly, the CFA of the four proposed measurement models based on the data of four groups (group 1: Environmental benefits of GMF narrated by an expert; group 2: Health benefits of GMF narrated by an expert; group 3: Environmental benefits of GMF narrated without an expert; and group 4: Health benefits of GMF narrated without an expert) was performed to assess the constructs’ validity and the goodness of the model fit using the recommended indices [84].

Details of all groups’ measurement models’ fitness indices are available in Table 3. Besides, the validity of all constructs was observed when their original constructs were loaded in all groups’ measurement models on AMOS. The item deletion remained within the limit of recommended omission for achieving model fitness (see Table 4).

### 4.3. Hypothesis Testing

The study applied the multi-group analysis approach to determine the measurement invariance between the groups, which ascertained whether the groups were significantly different [59]. In doing so, a model based on the multi-group factors was run. The results revealed that all groups have significant differences for all constrained paths: Chi-square difference = 1.71, degree of freedom difference= 4, and the differences were significant at *p* = 0.001; and for the unconstrained paths: Chi-square difference = 1.71, degree of freedom difference = 4, and the difference was significant for both constrained and unconstrained (all paths). The findings of the series of chi-square differential assessments showed that there was variation among the four experimental groups across the variables of this study. Thus, the study performed hypotheses testing (see Table 3). Similarly, the model fitness of all four structural models revealed the goodness of the fit (see Table 3).

After confirming the four structural models’ fitness (see Table 3), inferential statistical analysis and hypotheses testing were conducted. The research followed the stepwise approach by adding the variables of the study and the control variables in the model. Two hypotheses were proposed based on the direct influences, including H1: The knowledge and perceived risk reduction; and H3: Attitude towards GMF and acceptance of the GMF relationship with varying intensities across the groups. The results of the multi-group (SEM) revealed that the direct influence of knowledge on perceived risk reduction was significant across all groups: (1) group 1 (β = 0.22 and *p* = 0.01), (2) group 2 (β = 0.27 and *p* = 0.05), (3) group 3 (β = 0.09 and *p* = 0.01), and (4) group 4 (β = 0.07 and *p* = 0.01). Hence, H1 was accepted; ad messages framed with an expert opinion have greater influence regarding lowering the perceived risk (see Table 5). Furthermore, the direct influence of the attitude towards GMF on acceptance of GMF was also found to be significant across all four groups: (1) group 1 (β = 0.30 and *p* = 0.01), (2) group 2 (β = 0.52 and *p* = 0.01), (3) group 3 (β = 0.18 and *p* = 0.01), and (4) group 4 (β = 0.25 and *p* = 0.01). After adding the control variables, no significant influence of the control variables was found. Hence, H3 was accepted and verified that the acceptance of GMF was observed to be more positive for those exposed to ad messages framed with an expert opinion.

### 4.4. Mediation Analysis

Subsequently, the mediation hypothesis testing was investigated (H2), whereby the perceived risk reduction was posited as the mediating variable between the attitude towards GMF and knowledge linkage (in all four groups). To determine this, the study employed the hierarchal linear modeling (HLM) approach by using bootstrapping techniques suggested in the literature [85]. The same process of HLM was used in the four groups to compare the strength of mediation through multi-group analysis techniques. The findings presented in Table 6 reveal that the perceived risk reduction mediates the association between the attitude towards GMF and knowledge in all groups. The direct influence of the perceived risk reduction on the attitude towards GMF was identified across all groups: (1) group 1 (β = 0.39, *p* = 0.01), (2) group 2 (β = 0.47, *p* = 0.01), (3) group 3 (β = 0.32, *p* = 0.01), and (4) group 4 (β = 0.21, *p* = 0.01). An indirect influence of the perceived risk reduction was observed across all groups: (1) group 1 (β = 0.36, *p* = 0.01, and variance accounted for 46.6%), (2) group 2 (β = 0.41, *p* = 0.01, and variance accounted for 46.6%), (3) group 3 (β = 0.22, *p* = 0.01, and variance accounted for 46.6%), and (4) group 4 (β = 0.17, *p* = 0.01, and variance accounted for 46.6%). The direct influence of knowledge on the attitude towards GMF was also found to be significant across all four groups: (1) group 1 (β = 0.23 and *p* = 0.01), (2) group 2 (β = 0.34 and *p* = 0.04), (3) group 3 (β = 0.10 and *p* = 0.01), and (4) group 4 (β = 0.14 and *p* = 0.05). Therefore, based on the hierarchal linear modeling (HLM) rule of thumb, it can be concluded that partial mediation of the perceived risk reduction existed across all groups. Hence, H2 was approved and thee implications are discussed in detail in the discussion section.

### 4.5. Moderation Analysis

To examine the moderation influence of the natural preference for food across all groups, four models were examined (one for each group). The main influence of attitude towards GMF on acceptance of GMF was significant across all four groups: (1) group 1 (β = 0.30 and *p* = 0.01), (2) group 2 (β = 0.52 and *p* = 0.01), (3) group 3 (β = 0.18 and *p* = 0.01), and (4) group 4 (β = 0.25 and *p* = 0.01). Separate models were used to identify the interactional effect of PNF and AT. The direct effect of the influence of a preference for natural food on the acceptance to GMF was significant across all four groups: (1) group 1 (β = −0.19), (2) group 2 (β = −0.23), (3) group 3 (β = −0.08), and (4) group 4 (β = −0.21).

After adding the interactional term of (AT XPNF), it was found that the moderating effect of the preference for natural food was significant across all four groups: (1) group 1 (β = −0.13), (2) group 2 (β = −0.11), (3) group 3 (β = −0.16), and (4) group 4 (β = −0.24). The results presented in Table 7 support H4, in that the strength of the attitude and acceptance of GMF is a function of the preference for natural food. Therefore, it is shown that when individuals have a higher preference for natural food, they have a lower desire to buy or accept GMF.

## 5. Discussion

**Figure 2:**
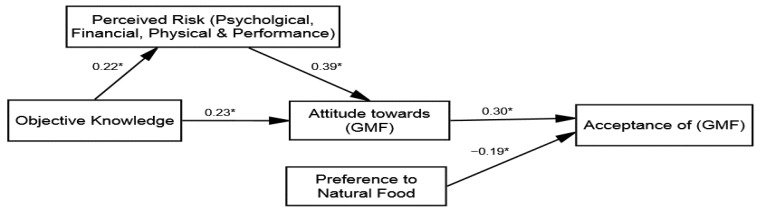
Structural model of group 1 (environmental message—expert opinion). * *p* ≤ 0.05.

The study used a quasi-experimental design (2 × 2) based on four groups of participants (exposed to different ad-messages) to validate the central idea that exposure to different ad-framed messages in the presence or absence of subject experts may have different implications across the variables involved in this mechanism. The study used the multi-group co-variance approach of SEM to validate the proposed hypotheses. This analysis approach helped to verify the hypotheses across all groups and confirmed the measurement invariance as well [86]. The study proposed H1, which is the direct influence of knowledge in reducing the perceived risk, to compare the effectiveness of an expert presence as well as the type of message (environmental vs. health benefits). The results (see Figure 2) revealed that knowledge directly reduces the perceived risk across all groups. These results supported SLT theory’s assumption regarding the efficacy of knowledge in diminishing risk perception [61,62]. Past studies also found that domain-specific knowledge helps decrease such risks about food products manufactured through modern food technologies, such as genetic modification, nano-food, and genetic engineering [63]. However, the comparison about the degree of the influence in reducing perceived risks across groups revealed interesting results both conceptually and managerially. Past studies reported that people have dread and unknown perceptions about GMF and have empirically tested the usefulness of improved knowledge [26,36,61].

**Figure 3:**
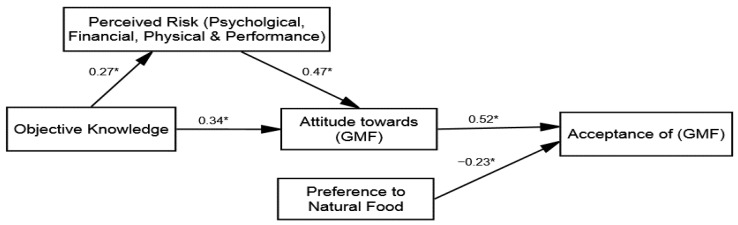
Structural model of group 2 (health message—expert opinion). * *p* ≤ 0.05.

Our results extend the body of knowledge and show that perceptions about GMF as a dread (risky activity) or based on unknown knowledge can be decreased (see Figure 3). To do so, ad messages may delineate the benefits to rectify most concerning issues (physical, psychological, etc.), such as health or environment, but from trusted experts. Likewise, the result of MG-CFA verified the substantial variance in the influence of the attitude on acceptance of GMF across the four groups. This is also consistent with past studies that affirmed that the public, in case of community or market acceptance, remain more mindful to choice regarding sensitive things, such as food [19,20]. However, interestingly, it was validated that the valence or frame of issues (health), which is considered more personal and can be more concerning to them, remained more effective (β = 0.52).

**Figure 4:**
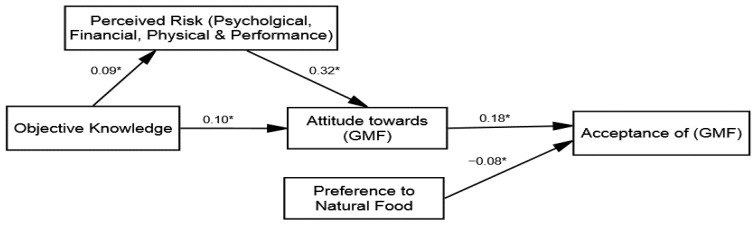
Structural model of group 3 (environmental message—no expert opinion). * *p* ≤ 0.05.

To this end, the literature has verified the assumption that individuals generally show more interest in the benefits and rely on cognitive shortcuts, such as an appealing message in terms of the source and action-related cues. Past studies also argue that people desire messages about technology-related products, specifically when they are uncertain about the acceptance [20,36]. Therefore, messages from well-respected sources can be an effective way to develop acceptance. The results of H1 about the direct influence of knowledge on perceived risk reduction (group 1 β = 0.22, group 2 β = 0.27, group 3 β = 0.09, and group 4 β = 0.07) are consistent with several psychological theoretical paradigms that describe how individuals observe threats and how they process risk information to act [20,39]. For instance, our results showed that participants that were exposed to the ad messages containing expert opinions experienced a greater reduction in perceived risks about GMF (see Figure 4). Previous studies on risk perception also explained this phenomenon, in that people generally perceive risk according to interpretations made from media content [52]. Afterward, mediation analysis was carried out to validate the assumption that perceived risks are the underlying mechanism defining the attitude intensity in the case of GMF. The result of the hierarchal linear modeling (HLM) revealed that the perceived risk reduction, which partially mediates the association between knowledge and attitude towards GMF across four groups, was varied. The literature has also shown that people are guided by their beliefs in determining their attitudes, and the results of the mediation analysis validate this populous notion [28].

**Figure 5:**
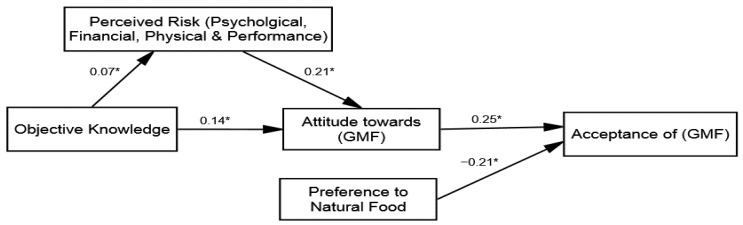
Structural model of group 4 (health message—no expert opinion). * *p* ≤ 0.05.

Conversely, one aspect of the mediation result is quite remarkable: the findings suggest perceived risk reduction is a stronger mediating factor (see Figure 5) among the participants who received health benefit-related information from an expert (reliable source). This is in line with the communication scholarship notion that people take a more cognitively active role when they are exposed to concerning issues, such as health, because they uphold more cognitive desires on such issues [20]. For this reason, past studies [44] also validated the central role of risk-related factors and our study has extended these results by testing the combined effects of the source and message to capitalize on the individual’s cognitive desire in developing a positive attitude towards GMF.

Furthermore, the study considered one crucial moderating social factor of a preference for natural food, which can define acceptance of GMF. The outcomes of the moderation of a preference for natural food were found to be significant across all groups (see Table 5). However, the moderation was inverse as expected based on the literature. Past studies also recommended that preferences for naturalness in several societies resulted in a lower desire to consume technologically produced food products [79,80]. Our results also revealed that participants across all groups showed a declining trend in terms of the inverse effect of natural preferences. However, this is a factor that can influence acceptance of GMF to a minute to a moderate extent when people receive information regarding the benefits from an expert they rely on and thus alter their overall attitude positively.

### 5.1. Theoretical Implications

This study endeavors to recognize the effective advertisement message strategy to endorse the necessity for an improved and manageable promotional campaign of GMF. Generally, advertisement messages focus on the targeted areas of behavioral or perpetual obstacles. Therefore, the literature was examined carefully to identify the prevailing issues that stated some obstacles and information processing issues in the context of GMF. For example, the scholar noted that the use of technologies, such as genetic engineering, to produce food products can be perceived as risky due to misperceptions [71]. In a similar vein, some studies affirmed that such safety-related issues prevail in society based on little or no domain-specific knowledge about scientifically innovated technology, such as GMF [28,69]. Hence, the literature suggests that reducing risk perceptions is an effective plan to modify individuals’ attitudes and acceptance. In this regard, communication scholarship provides implications regarding the influence of the message and the medium [50]. For instance, the research noted that well-designed ad messages can persuade individuals unless the selection of the message frame has been made deliberately.

The study empirically validates the notion of past behavioral change theories, such as the risk perception model and health belief, which describe perceived risk as imperative antecedents to form particular behaviors, whereby uncertainties are involved [52]. Similarly, risk information models, such as CMT, also emphasized that the outcome of information activity, such as promotional campaigns, relies on people’s evaluation of the risk. However, the information processing models (e.g., HSM) clarified that individuals’ perceived risk could be influenced by how the message has been framed [35]. Therefore, drawing on these theories, we empirically compared certain advertisement message frames based on their characteristics underlined in the literature. For example, information characteristics, such as (1) the frame utilized for unfolding risk (e.g., threatening), (2) message valence (e.g., positive or negative representation of the issue), (3) source of information (e.g., credible or sender itself); and (4) information format used (e.g., advertisement).

Theoretically, this study developed the hypotheses to contrast most of these information factors. For example, H1, H2, and H3 delineate the comparison between the source (expert vs. no expert opinion), valence, and frame (health or environmental benefits) used in an advertisement. The result of H1 supports the assumptions of CMT and trust determination theory that people trust and refer to expert opinions while making decisions [77]. Furthermore, the availability of expert opinions provides a better mechanism for forming an attitude that involves less cognitive labor. Likewise, the results of H2 provide empirical support for the behavioral change models emphasizing the significance of perceived perceptions. Correspondingly, H3 validated the standpoint of the risk perception attitude framework and provided further clarification in that when individuals have reduced perception of risks, attitude change can occur, which can lead towards acceptance of GMF usage [87,88]. Past theories, such as social amplification of risk theory (SART), attempted to relate societal factors and people’s procedural assessment of risks [68]. SART suggested that people’s societal response structure can outline their understanding of risks. Therefore, when people come across an uncertain situation, such as the adoption of new products, they try to evade such an adoption. The results of H4 support this notion and found an inverse moderating effect of the preferences for natural food in predicting the acceptance of GMF usage. These results also correlate with Pakistani societal and cultural preferences and in this way, the results suggest that the societal response structure has influenced people’s behaviors toward GMF.

To this point, it has been well established that how advertising illustrates information can influence its receivers’ perceptions and attitudes [33]. Similarly, unitization of news media content has been established in health-, environmental-, and promotion of cause-related issues. For example, a recent study authenticated the influence of message presentation categories of scientific knowledge-based health information on individuals’ perceived risk and attitudes towards GMF. However, the study relied on assumptions from previous theories, such as SLT and CMT, which recommend an understanding of the dual processing of the human mind (elaboration vs. cognitive shortcut). People rely on the role of domain-specific knowledge, for instance, to elaborate and reduce their risks. Although the presentation of an advertisement message (ad-framed) in this way helps to reduce the perceived risk, the message source’s credibility (experts) can also make a difference by providing effortless cognitive cues for action. Therefore, combining these two aspects led to the design of a quasi-experimental study to understand how varying framed types of ads can influence one’s knowledge, risk perceptions, and attitudes toward acceptance of GMF. In this way, the findings provide some interesting and novel solution-oriented directions for the future.

### 5.2. Managerial Implications

The current study provides managerial implications to develop effective marketing communication promotional campaigns to motivate people to adopt modern food technologies. Drawing on the recent food security issues, such as food shortage across the globe, it is time to examine effective means to promote the acceptance of food produced through more sophisticated technology. However, perceived risks and ignorance are important phenomena that can hinder food technology promotion among the masses. The study tested a theory-driven model, which suggested that advertisement messages using subject experts delineating the benefits can be an effective strategy to reduce the perceived risk, resulting in acceptance of GMF. GMF managers must consider four points while promoting the GMF, including (1) use of a credible source of information, (2) explanation of the performance and benefits of GMF to clarify misperceptions, (3) utilization of rationale appeals in messaging instead of relying on fear appeals, and (4) delineation of the practical aspects, such as personal (health) benefits, to reduce risk perception.

### 5.3. Limitations and Future Recommendations

This research used a positivistic approach and prior theoretical arguments to develop a quasi-experimental design, thus there are two main limitations and one replication suggestion. First, future studies should use a qualitative study to identify new phenomena and factors involved in addressing risk perception and acceptance of food technology. Second, future studies should use the randomization method in experiments to strengthen the internal validity, which was a limitation of the current study due to a lack of resources. Thirdly, a large-scale nationally representative survey should be carried out in the future to replicate the results of the study. This will provide generalizable suggestions for managers. Lastly, however, the current study provided experimental evidence and identified the influence of the types of messages and sources in predicting the acceptance of GMF, however, this was limited to the one media content (advertisements). In recent times, the media environment has gradually become more diverse and new means of communication have been introduced, such as social media. Therefore, future studies should investigate the diverse impacts that social media may have on the adoption of technology-based food products.

## 6. Conclusions

Owing to the emerging challenges of climate change, global food insecurity and malnutrition are prominent issues for the 21st century. As the population increases, ensuring the globe has plentiful nutritious food is a problematic task. However, contemporary research has provided alternatives to traditional agriculture, such as the growth of GMF, to reduce global food insecurity. GMF enhances the nutritional qualities of food to combat malnutrition by consuming fewer agricultural inputs and saving resources, such as water. In developing countries, decades of controversy over innovative food technology-based products is also a challenge to enhancing their acceptance among the general public. Therefore, this study has provided useful and practical findings about how these challenges can be tackled through a well-designed communication campaign. The study concludes that expert involvement in advertisement messaging is substantial while promoting GMF.

## Figures and Tables

**Table 1:** Demographic Attributes.

Demographic	Frequency	Percentage
Gender		
Male	168	58.5
Female	132	41.5
Education		
Primary	48	16.0
High School	27	9.0
Undergraduate	42	14.0
Master and Above	150	50.0
Un-educated	33	11.0
Locality		
Urban	313	78.2
Rural	87	21.8
Age		
18–30	152	50.7
31–45	109	36.3
46–above	39	13.0

**Table 2:** Descriptive and Pearson correlation statistics.

**Group 1**	**Mean**	**PRR**	**OK**	**AT**	**PNF**	**AC**	**Group 2**	**Mean**	**PRR**	**OK**	**AT**	**PNF**	**AC**
PRR	2.35	1					PRR	1.76	1				
OK	3.37	0.44 *	1				OK	3.61	0.51 *	1			
AT	3.16	0.32 *	0.37 *	1			AT	3.72	0.53 *	0.65 *	1		
PNF	2.10	0.24 *	0.39 *	0.41 *	1		PNF	2.39	0.49 *	0.76 *	0.65 *	1	
AC	4.04	0.36 *	0.56 *	0.21 *	0.43 *	1	AC	3.92	0.42 *	0.63 *	0.43 *	0.68 *	1
**Group 3**	**Mean**	**PRR**	**OK**	**AT**	**PNF**	**AC**	**Group 4**	**Mean**	**PRR**	**OK**	**AT**	**PNF**	**AC**
PRR	2.71	1					PRR	2.59	1				
OK	2.86	0.16 *	1				OK	1.89	0.27 *	1			
AT	2.52	0.26 *	0.23 *	1			AT	1.78	0.41 *	0.65 *	1		
PNF	2.63	0.19 *	0.09 *	0.13 *	1		PNF	2.45	0.32 *	0.76 *	0.65 *	1	
AC	2.57	0.18 *	0.22 *	0.19 *	0.27 *	1	AC	2.21	0.24 *	0.32 *	0.20 *	0.14 *	1

(Each group *n* = 75 *n* = 300) * *p* ≤ 0.05. PRR = Perceived Risk Reduction, OK = Objective Knowledge, AT = Attitude, PNF = Preference for natural food and AC = Acceptance of GMF.

**Table 3:** Confirmatory factor analysis.

**Measurement Models**	**x^2^/df**	**GFI**	**TLI**	**IFI**	**CFI**	**RMSEA**
Group 1	1.81	0.95	0.96	0.97	0.95	0.035
Group 2	2.03	0.91	0.91	0.92	0.93	0.030
Group 3	2.63	0.98	0.95	0.97	0.96	0.041
Group 4	2.19	0.93	0.90	0.92	0.98	0.038
**Structural Models**	**x^2^/df**	**GFI**	**TLI**	**IFI**	**CFI**	**RMSEA**
Group 1	2.09	0.96	0.95	0.91	0.98	0.034
Group 2	1.67	0.97	0.91	0.93	0.95	0.050
Group 3	3.41	0.98	0.98	0.90	0.94	0.032
Group 4	4.12	0.93	0.92	0.97	0.91	0.037

GFI = Goodness of Fit Index, TLI = Tucker–Lewis Index, IFI = Incremental Fit Index, CFI = Comparative Fit Index and RMSEA = Root Mean Square Error of Approximation.

**Table 4:** Validity statistics and standardized weights.

Items	Group 1				Group 2				Group 3				Group 4			
	α	CR	AVE	W	α	CR	AVE	W	α	CR	AVE	W	α	CR	AVE	W
PRR1	0.83	0.92	0.61	0.89	0.91	0.93	0.64	0.76	0.77	0.87	0.69	0.76	0.74	0.89	0.55	0.82
PRR2				0.76				0.69				0.89				0.71
PRR3				0.65				0.84				0.71				0.77
PRR4				0.81				0.77				0.74				0.64
PRR5				0.94				0.89				0.64				0.69
PRR6				0.72				0.86				0.34 *				0.73
PRR7				0.68				0.75				0.78				0.81
PRR8				0.73				0.83				0.63				0.72
OK1	0.85	0.92	0.68	0.91	0.89	0.94	0.71	0.93	0.74	0.91	0.62	0.78	0.79	0.89	0.60	0.86
OK2				0.86				0.69				0.81				0.63
OK3				0.78				0.93				0.84				0.79
OK4				0.81				0.86				0.88				0.82
OK5				0.74				0.83				0.67				0.71
OK6				0.84				0.79				0.74				0.80
AT1	0.83	0.87	0.69	0.90	0.93	0.90	0.72	0.92	0.71	0.83	0.63	0.82	0.76	0.86	0.64	0.85
AT2				0.65				0.84				0.77				0.73
AT3				0.89				0.86				0.23 *				0.78
AT4				0.85				0.77				0.79				0.84
PNF1	0.88	0.89	0.63	0.67	0.82	0.86	0.62	0.74	0.79	0.83	0.60	0.89	0.70	0.80	0.62	0.91
PNF2				0.82				0.71				0.68				0.76
PNF3				0.76				0.86				0.75				0.69
PNF4				0.91				0.83				0.77				0.43 *
AC1	0.86	0.89	0.72	0.82	0.92	0.90	0.79	0.92	0.73	0.79	0.56	0.71	0.80	0.84	0.67	0.89
AC2				0.84				0.81				0.69				0.77
AC3				0.90				0.93				0.84				0.80

PRR = Perceived Risk Reduction, OK = Objective Knowledge, AT = Attitude, PNF = Preference for natural food and AC = Acceptance of GMF, W = item weights, CR = Composite Reliability, AVE = Average Variance Extracted and * = removed items.

**Table 5:** Hypothesis testing.

Direct Influence	PRR←OK	AC←AT
Group 1: Environmental message—Expert opinion	0.22 *	0.30 *
Group 2: Health message—Expert opinion	0.27 *	0.52 *
Group 3: Environmental message—no Expert opinion	0.09 *	0.17 *
Group 4: Environmental message—no Expert opinion	0.07 *	0.25 *

* *p* ≤ 0.05.

**Table 6:** Meditation results.

Mediation Models	Direct Effect β	Indirect Effect β	Meditation
Group 1: Environmental message—Expert presence	0.23 *	0.36 *	Partial
Group 2: Health message—Expert presence	0.34 *	0.41 *	Partial
Group 3: Environmental message—Expert Absence	0.10 *	0.22 *	Partial
Group 4: Environmental message—Expert Absence	0.14 *	0.17 *	Partial

β = Standardized Regression Weight and * *p* ≤ 0.05.

**Table 7:** Moderation results.

Stepwise Moderation	Results
Group 1: Environmental message—Expert presence, Dependent Variables: Acceptance of GMF	
Step 1: Independent Variables: Attitude	0.30 * (4.15)
PNF	−0.19 * (4.87)
R^2^ Step 2: Moderator: AT × PNF	0.39
	−0.13 * (5.12)
R^2^	0.32
ΔR2	0.07
Group 2: Health message—Expert presence, DV: Acceptance of GMF	
Step 1: Independent Variables: Attitude	0.52 * (3.24)
PNF	−0.23 * (3.96)
R^2^ Step 2: Moderator: AT × PNF	0.62
	−0.11 * (4.45)
R^2^	0.47
ΔR2	0.15
Group 3: Environmental message—Expert Absence, DV: Acceptance of GMF	
Step 1: Independent Variables: Attitude	0.18 * (6.73)
PNF	−0.08 * (3.65)
R^2^ Step 2: Moderator: AT × PNF	0.31
	−0.16 * (5. 94)
R^2^	0.21
ΔR2	0.10
Group 4: Health message—Expert Absence, DV: Acceptance of GMF	
Step 1: Independent Variables: Attitude	0.25 * (4.15)
PNF	−0.21 * (4.87)
R^2^ Step 2: Moderator: AT × PNF	0.18
	−0.24 * (5.192)
R^2^	0.14
ΔR2	0.04

Note. The values in parentheses represent t statistics. Entries are random effects with a robust standard error. AT = Attitude, PNF = Preference for natural food, DV= Dependent Variable, R^2^ = proportion of variance explained by the antecedent in both model 1 and 2, * *p* ≤ 0.05.

## Data Availability

The data that support the findings of this study are available from the corresponding author upon reasonable request due to ethical and privacy restrictions.

## Appendix A

1. Ad-content for Group 1: Environmental benefits of GMF narrated by an expert

Narrated by Expert in the display: There is a Scientific community of climate, food technology, and environmental experts on the safety of Genetically modified foods. The scientists who have researched on GMF field agree on the position that “the GMFs currently on the market are eco-friendlier and safe for the environment than their non-GMF counterparts”. They are more beneficial for our planet as they save natural resources. Hence, the use of GMF can help to combat climate change and global food insecurity.

2. Ad-content for Group 2: Health benefits of GMF narrated by expert

Narrated by Expert in the display: There is a Scientific community of public health, food technology, and medical experts on the safety of Genetically modified foods. The scientists who have researched on GMF field agree on the position that “the GMFs currently on the market is a better source of nutrition and safe for health than their non-GMF counterparts and does not lead towards food allergies”. They are more beneficial for human health as they provide contains more nutrients and save from malnutrition. Hence, the use of GMF can help to combat malnutrition and global food insecurity.

3. Ad-content for Group 3: Environmental benefits of GMF narrated without an expert

Display: There is a Scientific community of climate, food technology, and environmental experts on the safety of Genetically modified foods. The scientists who have researched on GMF field agree on the position that “the GMFs currently on the market are eco-friendlier and safe for the environment than their non-GMF counterparts”. They are more beneficial for our planet as they save natural resources. Hence, the use of GMF can help to combat climate change and global food insecurity.

4. Ad-content for Group 4: Health benefits of GMF narrated without an expert

Display: There is a Scientific community of public health, food technology, and medical experts on the safety of Genetically modified foods. The scientists who have researched on GMF field agree on the position that “the GMFs currently on the market is a better source of nutrition and safe for health than their non-GMF counterparts and does not lead towards food allergies”. They are more beneficial for human health as they provide contains more nutrients and save from malnutrition. Hence, the use of GMF can help to combat malnutrition and global food insecurity.
